# “Fun for some, terrible for others” Gender, risk and responsibility in young people's stories about alcohol consumption

**DOI:** 10.1177/14550725251350528

**Published:** 2025-06-30

**Authors:** Annadís Greta Rúdólfsdóttir, Ragný Þóra Guðjohnsen

**Affiliations:** 64435 University of Iceland, Reykjavík, Iceland; University of Iceland, Reykjavík, Iceland

**Keywords:** gender, responsibility, risk, shame, young people’s alcohol use

## Abstract

**Aim:** This paper explores how gender, risk, responsibility and shame feature in young people’s (aged 18–20 years) stories about alcohol consumption with peers in Iceland. **Methods:** The data consist of 73 stories generated through the story completion method. The participants were presented with a story stem and invited to complete it. The stories were analysed using reflexive thematic analysis. The stories were selected to provide insights into the affect underpinning gendered bodies and certain cultural scenarios. **Results:** Alcohol consumption was presented not only as fun, but also as a way to calm nerves around expressing sexuality. Anxieties and regrets about alcohol consumption were gendered. Young female characters were shamed for drinking too much and portrayed as more at risk of coming to harm because of sexual violence. However, there were no signs of slut-shaming. To deal with the risks, they took measures such as being accompanied by a responsible sober friend. Parents were conspicuous by their absence, and adults rarely featured in the stories. **Conclusions:** The research provides insights into how young people’s relations with alcohol are mediated by gender. The risks the participants identified in their fictional accounts should be considered in policies and pedagogical attempts to reduce harmful alcohol consumption practices. Schools should play a greater role in increasing young people’s knowledge and understanding of alcohol and drug use and the social, behavioural, emotional and moral aspects associated with it.

## Introduction

Young people aged between 18 and 25 years are at the cusp of adulthood in the twilight zone between being a teenager and an adult. Sometimes referred to as emerging adults (Arnett, 2000), they are in an age when they are figuring out what aspects of adult behaviour they want to make their own, including consumption of alcohol. Intoxication has played a significant role in the current Icelandic drinking culture, which can be seen to have developed in the latter half of the 20th century (Di Genova, 2024). At the beginning of the 21st century, concerns about heavy alcohol consumption among young people started to grow. With joint preventive efforts of parents, schools and the leisure sector, teenage drinking slowly decreased (Sigfusdottir et al., 2020). Lifetime intoxication of 15-year-old students dropped from 67% in 1995 to 14% in 2014 (Haraldsson et al., 2022). However, alcohol drinking and intoxication have remained much higher in upper secondary school (Guðjohnsen et al., 2024), and drinking with friends as part of going out together is a major part of adult nightlife in Iceland, now classified among high alcohol consumption countries such as Finland, Ireland, Luxembourg and Malta (Correia et al., 2024). During the period of increasing national alcohol consumption, control policies in Iceland weakened significantly, despite previously being regarded as exemplary among OECD countries (Babor et al., 2023).

Despite an overall decline in teenagers and young people’s alcohol consumption in Iceland, research suggests that alcohol still features significantly in their night-time recreational activities (ESPAD Group, 2020). Studies show that, in 2018, 26% of 16–18-year-old students and 50% of students aged between 18 and 20 years reported that they had been drunk at least once in the past month (Guðmundsdóttir et al., 2020). In other words, young people seem to be easing themselves into an adult culture and developing lifestyles where the decision to drink or refrain from alcohol is important (Nicholls, 2019).

Both social and health risks as well as the pleasures of alcohol consumption are well documented (Hunt et al., 2022a). Numerous research studies have also shown that expectations and norms in relation to alcohol consumption may vary in terms of gender (Bogren, 2019; Day et al., 2004; Dempster, 2011; Measham, 2002) and other categories of difference, such as social class (Bogren, 2019; Griffin et al., 2013; Lennox et al., 2018), ethnicities and sexualities (Bernhardsson & Bogren, 2012), which in turn can contribute to an evidence base that frames public understanding and governance of alcohol-related behaviours (Austen, 2009).

In this paper, we focus on discursive and affective constructions of alcohol consumption and its associated risks as represented in fictional stories generated specifically for this study (Braun et al. 2019; Clarke et al. 2017). Students (18–20 years old) were presented with a story stem and invited to complete a written story about young people and alcohol consumption. The appeal of this method rests in that participants get to write about potentially sensitive topics without making themselves central to the stories. At the same time, they need to draw on their socio-cultural knowledge for completing the sense-making of the stories. We draw on the work of Wetherell (2012) and Ahmed (2014) who point out that social meaning systems, such as discourses of knowledge, both produce and carry affect. Discourses of knowledge affectively pull in subjects and regulate them through their emotions and desires (Orgad & Gill, 2022) such as shame and wanting to belong or fit in. They set out the norms for how we should feel and perform and, as such, are clearly linked to social values and imply judgement (Wetherell, 2012). We argue that the stories generated through our research provide insight into young people’s ideas not only about intoxication, but also the affects mobilised when outlining the consequences of drinking alcohol. In this regard, our study contributes to research that is increasingly paying joint attention to the discursive and the affective in empirical work on young people’s entries into cultures of intoxication (Frank et al., 2020).

What this analysis contributes to existing research, is the focus on how responsibility and shame feature in young people’s (aged 18–20 years) stories about alcohol consumption with peers. We argue that shame is particularly interesting in the Icelandic context because it has been a central concept in young feminist movements (Rúdólfsdóttir & Jóhannsdóttir, 2018) and public discussion about gender-based violence (Pétursdóttir & Rúdólfsdóttir, 2021). The research questions guiding our work are as follows: How does gender feature in young people’s stories about alcohol consumption and risks in their social lives? How do shame and responsibility feature in these stories?

### Alcohol, gender and risk

We use the concept risk quite loosely to refer to socially constructed understandings of how a persons’ actions, in this case alcohol consumption, might impact negatively on people’s lives in the future. Alcohol intoxication has been considered to present risks both individually and socially and therefore there have been attempts to regulate consumption of alcohol. These regulations in turn are embedded within cultures of intoxication and cultures of sexualities and have been highly gendered (Hunt et al., 2022b). Depictions of young women’s intoxication constitute a case in point. Public mainstream representations of alcohol consumption have tended to align with gendered discourses (Månsson & Bogren, 2012; Nicholls, 2019). In terms of risk, young women’s use of alcohol has been more readily presented as having negative consequences in public discourses than young men’s consumption of alcohol. In this regard, Brown and Gregg (2012) pointed out how public health campaigns have framed young women’s excessive drinking as highly risky and likely to result in shame and regret. Their drunken activities have been framed in terms of vulnerability, where they can become the victims of preying men (Månsson & Bogren, 2012). In contrast, men’s drinking has been related to masculine identities and adventure (Hunt & Antin, 2019), thus evoking feelings such as fun and excitement. Men have been expected to seek sexual experiences (Grazian, 2007; Hunt & Antin, 2019) and find pleasure in drinking excessively (Dempster, 2011), whereas the risk of shame is always on the horizon for young women. The practices around alcohol consumption are thus embodied and have an affective force in the sense that they attract and repel emotions that in turn shape what activities are considered possible.

The cultural meanings of gender and alcohol consumption are neither static, nor immutable and, at times, these can be contradictory. Traditional depictions of women’s consumption of alcohol as posing risks to their sexuality and moral reputation (Bogren, 2019; Brown & Gregg, 2012; MacLean et al., 2018) contrast with ideas that young women consume alcohol for pleasure and as part of expressing their sexualities, lifestyle choices and identities (Bogren et al., 2023; Griffin et al. 2013). These mixed messages call forth a difficult balancing act for young women. That, on the one hand, they are letting their hair down and open to sexual liaisons, yet, on the other hand, they must bear in mind ideas about respectability so that they do not risk their moral reputation and thereby shame (Hutton, 2020). In this regard, Ahmed (2014) argues that an embodied feeling that we find hard to talk about, works as an intense form of social control. Riley et al. (2023) further points out that shaming as a social process requires another individual to do the shaming. The social group that young people are part of is thus important for signalling the extent to which a young person’s behaviour while intoxicated is shameful or not. This is not always a straightforward issue. As mentioned in Hunt et al. (2022b), study factors such as levels of intoxication may call forth different gendered sexual scripts and moral standards in young people’s peer groups. Low levels of alcohol intoxication were considered to work as a lubricant in social and sexual relations, whereas high levels of intoxication were considered to blur or distort signals in relation to sexual consent. Young women are being left with the blame and shame for sexual violence that they are subjected to as they shoulder the responsibility of having become so intoxicated that they could not express whether they consented to sexual relations (Petersen et al., 2022). These gendered double standards also come across clearly in the study by MacLean et al. (2018), which portrayed how the behaviour of highly intoxicated young women elicited mixed feelings. Young women’s drunken antics were potentially funny but caused disgust when perceived as out of control and sexually inappropriate. In contrast, heavily intoxicated young men were considered threatening but did not trigger the same visceral repulsion. In other words, the moral risks of alcohol consumption have been higher for young women (Atkinson & Sumnall, 2016).

Importantly, the way in which young people’s intoxication is made sense of also depends on the social circumstances and company they find themselves in. Various studies show how alcohol consumption affirms young people’s personal relationship with a friendship group (Abrahamson, 2004; Jensen et al., 2019). The social role alcohol plays in young people’s lives comes across in the importance drinking stories have for friendship groups (Conroy & MacLean, 2019; Tutenges & Sandberg, 2013). They have an entertainment value for the group as a whole and young people even get drunk to be able to share humorous stories (Griffin et al., 2009). In this respect, Rolando et al. (2015) found that young women film each other to capture the convivial and fun aspect of drinking, whereas the study by Brown and Gregg (2012) showed that young women share risky and potentially regrettable events with one another on Facebook as part of their ongoing narrative of what they were up to as a group. Alcohol consumption and the stories told about what happened thus affirmed young people’s belonging to a particular group and were not used to shame them. In this sense, the shared stories were not just about the individual but, importantly, about the peer groups bonding together (Fjær, 2012; Tutenges & Sandberg, 2013). Indeed, as argued by Conroy and MacLean (2019), alcohol appears as a key ingredient in constituting friendship.

The peer group is also important in the sense that it may collectively attempt to navigate risks of excessive drinking. Friendship groups may egg individual members on when it comes to drinking, although they also take on the responsibility to ensure individual members do not come to harm (Conroy & MacLean, 2019). What we read out of these studies is that group members may shield each other from affects such as shame that harm especially young women’s reputation. This underscores the entanglement of discourses and affect and the joint role they play in individuals’ surveillance of themselves and other, whether it is to avoid discomfort and safeguard reputations or judge others. Importantly, shame has also been a key concept in young feminist movements in Iceland (Rúdólfsdóttir & Jóhannsdóttir, 2018) and MeToo (Pétursdóttir & Rúdólfsdóttir, 2021), where an emphasis has been placed on removing shame from the bodies of survivors-victims of sexual violence, including when drunk, and sticking it to where it belongs (i.e., the perpetrators). Attempts to shame are in other words increasingly being called out and criticised as a form of social control.

## Methods

The study, which received a positive review from the University of Iceland Ethics Committee, employs a qualitative approach using the story stem method (Braun et al. 2019). Before participating in the research, all participants were provided with information about the study and gave their fully informed consent online. The participants were never asked for their names, and the researchers had no access to information that could identify the participants.

Two recruitment strategies were employed to engage participants. First, two advertisements were posted on Facebook directed at young people aged between 18 and 20 years. For that, we paid a small fee. In the advertisement, information was provided about the research objectives and participants’ right to anonymity. Then a link led those who wanted to participate to the actual study. Second, various upper secondary schools were contacted in both Reykjavík and other urban areas in Iceland and they were asked to inform their students about the study. This task was made easier by the fact that Iceland is quite small with a population of less than 400,000 and we had good connections to the upper secondary schools through contacts.

In total, 72 participants took part in the study, of whom 58 identified as female (F), 13 as male (M) and one did not identify any gender. They represented both Reykjavík and other urban areas in Iceland and were enrolled in a total of 22 upper secondary schools spread across the country. We actively tried to involve more participants identifying as male but were unfortunately not successful.

Participants were presented with a story completion task online and were directed to a webpage where they were randomly assigned one of three story stems. The design and theoretical approach of the stories were rooted in giving young people opportunities to write about potentially sensitive topics related to alcohol drinking (e.g., illegal activities), without making themselves central to the stories (Clarke et al., 2017). The stories were all identical, except for the main characters alternatively being girls/boys/friends. This allowed comparisons of the stories created in terms of the gender of the main protagonists. The stems were as follows:

The girls/boys/friends sat together and discussed the events that occurred on the previous night. They had been invited to a party where alcohol was offered. What happened then?

In the instructions, students were asked to continue with the story and expand further on the following in the stories: (a) Describe the girls/boys/friends. (b) Where did the events take place? (c) Did they have a good time? Apart from that they were not given guides on what to write about. They were asked to spend at least 10 minutes writing the stories but were not given instructions of how long the stories had to be.

Although such stories are fictional, the theoretical approach rests on the benefits they provide by offering insights into the knowledge, affect and assumptions participants draw upon when making sense of particular social issues.

The stories were analysed in line with the guidelines of Braun and Clarke (2021) for reflexive thematic analysis. This involved careful reading and coding of the material while keeping our research questions in mind. The coding was inductive and focused on representing meaning as communicated through the stories related not only to how gender features in young people’s stories about alcohol consumption and risks in their social lives, but also to how shame and responsibility features in these stories.

The analysed materials amounted to approximately 12,000 words. The provided stories were dense in meaning but ranged from one or two sentences to complex stories in several paragraphs. Most stories were about one paragraph or around 200 words.

Because we were interested in knowledge and values related to gender, we especially looked for whether the stories referred to gendered knowledge frameworks and discourses for this analysis, especially in relation to constructions of risk. We explored whether these stories had affective subtexts, for example, in relation to what kinds of emotions the stories evoked and to what story characters and bodies they were attached. We then carefully went through the codes and constructed potential themes that we thought would shed light on the research question.

The extracts which were translated from Icelandic to English were chosen to underscore our analysis.

## Results

The stories are made up by the participants and should thus be regarded as fictional. As such, they span a spectrum ranging from the light-hearted and humorous to the dark and serious; from the cozy and mundane to the grotesque and scary. However, the participants placed the main protagonists of their stories in a social situation that most of them seemed to know well: going to the secondary school class parties or school dance organised for students or to a party organised in an urban setting. There were also stories that followed groups of friends downtown. In other words, the stories highlighted shared knowledge about the social settings of the alcohol consumption and possible consequences of drinking. As the quote in the title of this article conveys, “It was fun for some and terrible for others” (M), the participants expressed ambivalent feelings about alcohol consumption. The overriding experience sought with alcohol in the stories was that of fun but, as MacLean (2016) points out, the enhanced affective mood that would be considered part of alcohol consumption generates social opportunities and risks. Many of the stories also had a moral message because they outlined in quite strong terms the gendered risks involved in drinking alcohol.

Based on our analysis, we discuss three themes and analyse in the following sections: (i) The imperative to have fun: Sexual and social situations navigated by alcohol. (ii) The risk of losing control. (iii) Dealing with moral dilemmas and issues of guilt and responsibility.

### The imperative to have fun: sexual and social situations navigated by alcohol

The first theme, *The imperative to have fun: Sexual and social situations navigated by alcohol*, was interesting because it cast alcohol as the solution to overcoming personal limitations in terms of shyness and making yourself available to social or sexual experiences. Alcohol was not strictly necessary as a facilitator for generating fun evening with friends but was always present in stories where those evenings led to sexual situations/relationships. Young story characters overcome with anxiety, gained the courage to chat up their object of interest when intoxicated, and be more open about their feelings and experiences with others. The affective state of comfort (Herold & Hunt, 2020) gained with the aid of alcohol enabled the story characters to talk to the person whom they fancied or just act on their desire to have sexual relations with someone when intoxicated. As in the case of the study by Jensen et al. (2019), here, drinking and flirting was mostly placed in a heterosexual context, but alcohol gave permission for sexual liaisons. Experiences of intoxication were in turn described as binding the peer groups together because they stimulated fun stories and shared memories that mattered.

In the story extract below, the protagonist Gunnar found courage in his intoxication and managed to overcome his personal limitations and have sexual relations with a girl. That not only pleased him, but also his friends in the story as well. Interestingly, the author stated that the boys drank with the sole intention “to get laid”. The story is in line with traditional ideas about the sexual prowess of masculinity (Grazian, 2007). Here Gunnar not only goes home with a girl, but she is someone he has never met before:

Gunnar, the shy type, went home with a girl whom he had never met before, only seen her on Instagram. The group was delighted as Gunnar usually didn’t do things like that (…). The others continue to enjoy life with a beer and some gossip. They only have one aim – to get laid – and that is the only reason they drink. (M)

Stories that focused on sexual intentions were not limited to those of young men. In some stories where the story protagonists were young women, it was described how fun kissing and drinking went hand in hand: “they talked about how much fun they had had and what boys they had kissed” (F). This fits with several research findings where young women reported that they found it easier to flirt when drunk (Abrahamson, 2004; Jensen et al., 2019; MacLean et al., 2018). In the stories we collected, the level of intoxication did matter as Hunt et al. (2022b) noted, but even high levels of intoxication were described as fun because it provided the main story character the company of friends:

He asked his friend whether they shouldn’t just do some shots. “Yes, yes, why not”. There was a school dance afterwards and better to be quite drunk if it should so happen that they would snog someone afterwards. (F)

In the stories, social media had the role to preserve memories of drunken events presented for entertainment that otherwise could be lost. In one of the stories in our sample, the main protagonist’s drunken antics was preserved on social media. In the story, Þórunn snogged a friend. No judgment or shame was passed on that action, but the event is saved for social media:

Have you seen this? Was I really that drunk? I don’t remember anything. Karitas and Eyrún took up their phones and looked at the story. There they could see Þórunn snogging Jón while Helgi calmly took their photo. (F)

In the story, the character Þórunn did not remember having snogged Jón but her exclamation “was I really that drunk?” seemed to be offered as an explanation for her actions. The story character was not by herself but surrounded by friends and no shame or regret is described. Tutenges and Sandberg (2013) note how important social media has become for documenting transgressions and playing a part in building up a narrative of what happens during drinking sessions. The evening was also fun because the end product was a good story. As alcohol increasingly became the star of the stories of a night out, the narratives became more intense and interesting. Indeed, some authors indicated that it was the anecdotes told that made becoming intoxicated interesting and provided spice to otherwise uneventful lives:

They (young women) pretend they are more drunk than they are. (…) It is not really about having fun but doing something big that they can talk about, that matters and makes their lives seem exciting. (F)

This is in line with the findings by Tutenges and Sandberg (2013) showing that a repertoire of drinking stories is valuable to young people. The presence of alcohol gave the stories an affective intensity or as stated in one of the stories: It imbued the story characters “normally dull lives” (F) with meaning. The protagonists even pretended to be more intoxicated than they were, aiming to be seen as someone fun and entertaining. The story implies that certain actions require alcohol.

This theme is in line with the findings from several research studies that show how important alcohol is considered in giving licence to find sexual partners and we see no shame attached to these actions (Abrahamson, 2004; Jensen et al., 2019; MacLean et al., 2018). Also portrayed is how alcohol consumption was seen as part of a bigger social event, binding a group together through their shared experiences and pleasures. The stories that friends shared were considered the most important products of the drinking sessions. The fun continued through the stories told. In those stories, the participants described alcohol consumption as a pleasurable friendship practice rather than an individual experience (MacLean et al., 2018; Niland et al., 2013).

### The risk of losing control

The second theme in the stories was *The risk of losing control* as high levels of intoxication and associated risks were frequently reported. The risks that the young people associate with alcohol drinking and intoxication have also been the foundation of alcohol prevention campaigns in Iceland, aimed towards decreasing drinking and increasing awareness of the risks affiliated with excessive alcohol consumption (Di Genova, 2024). The stories in which the characters were highly intoxicated played out in quite gendered ways and had different affective subtexts. These characters were also often described as younger students in the first year of junior college who still had not learned how to hold their drink. Risk played out in gendered ways. We saw instances of young women being shamed for drinking underage but no such instances when the characters were young men. In stories where characters were incapacitated by alcohol, young men were mostly portrayed as being at risk to themselves whereas young women faced risks from mostly male strangers with bad intentions.

Some of the stories described drunk characters who had passed out, covered in vomit or vomiting. MacLean et al. (2018) note how exposure to other people's body fluids is experienced as disgusting and vomiting in public as particularly repulsive. The stories in our data sets describing highly intoxicated story characters who were vomiting included both young men and women. However, humorous stories with grotesque descriptions geared to evoke disgust were predominantly about young men:

Most memorable is one of the younger (boys) who passed out as always in the bathroom, except this time, in the shower, wrapped up in the shower curtain covered by his own vomit. (…) The guy who passed out from drinking thought it was fun and was going to repeat it every weekend. The girl who vomited felt very ashamed and bad about what happened. (F)

In the preceding extract, the account of the young man who passed out from drinking was humorous; he was going to do it again, whereas the young woman was consumed with bad feelings and shame at having vomited. The implication is that heavy drinking, and its outcome is more acceptable for young men. As Griffin et al. (2009) pointed out, young men’s overindulgence of alcohol has been perceived as part of determined excess, whereas over-indulgence has been considered unfeminine and shameful (Bordo, 2003; Hunt et al., 2022b). This comes across clearly in the extract below where an encounter between a young woman and her love interest (older young man) is described:

He sat beside her, put his hand on the back of the sofa and asked her what she was doing in a party. She had always been this good girl, not involved in any nonsense and no one would have thought that she had started to drink this young. (F)

In the extract, the female character is shamed for not being a “good girl” by the young man she wanted to impress. She should not be in the party and should not be drinking underage. This is in contrast with other story lines in the data that outline how going out and having a drink is fun and enjoyable for young women. In itself, it underscores the mixed messages young women receive about consuming alcohol.

There were a number of stories about drinking that left the story character completely comatose. Most of these stories were about young women. Exceptions to this pattern were two stories that described young men who drank too much alcohol and ended up with alcohol poisoning: “One of them had such bad alcohol poisoning that he lost consciousness and was fetched by an ambulance” (F). However, in these stories, the young men are described as risking their lives not making a moral faux pas with their indulgence.

The stories about comatose young women sensate with fear and anxiety. Several stories implied or described rape scenes with young drunk women as victims where the perpetrators were men usually older and often characterised as strangers:

They had all been quite nervous (…) Guðrún had a crush on Kári … smiled … the kids danced, laughed, and had a great time (…). It wasn't until eleven o’clock, that three unknown older boys came out of the blue and everything started to take a turn for the worse. (F)

The stories thus followed traditional storylines where women are preyed on by men (Månsson & Bogren, 2012). The readers are presented with the bodies that predators could use as they wanted. However, despite the anxiety described in these stories and the focus on the vulnerability of young women, we did not see any overt signs of slut-shaming. The culprit was always the perpetrator who was usually described as a stranger taking advantage of the young women who they violated:

She had no energy and just “let” him kiss her even though she didn’t want him to. She just could not push him away. (F)

Risky situations that were considered to compromise the protagonists in the stories were described as causing regret and anxiety. The stories portrayed how the story characters worried about losing control of their agency. These stories contained many references to the vulnerability discourse (Bogren & Månsson, 2014) and there was a clear undercurrent of anxiety in stories about drunk young women. However, the stories are more about harm and most of the stories on sexual violation are not told from the perspective of the victim herself but rather story characters who are friends and worry about what is happening. What we also detected was the anxiety that bound itself to young women's bodies, their bodies were at risk of harm, whereas stories about drunk young men drew more on humour. Violators are described as men who take advantage. We did not see attempt to slut-shame young women in the stories and the possible reason may be a recent feminist awakening amongst young women in Iceland who have stressed that shame should be moved from victims of violence to the perpetrators (Rúdólfsdóttir & Jóhannsdóttir, 2018). However, the idea that young women may be at risk of harm still generates fear and anxiety that gravitates towards the young female body.

### Dealing with moral dilemmas and issues of guilt and responsibility

The third theme, *Dealing with moral dilemmas and issues of guilt and responsibility*, connects with the theme, *The risk of losing control*, but centres around activities taken to avoid perceived risk. The crux of the stories were often moral dilemmas and anxiety about doing the right thing with a focus on what it meant to be responsible. The stories depicted how the risks around alcohol consumption were not just the responsibility of the story character who had consumed alcohol but the friendship group as a whole. This was especially the case when the main characters were young women. Interestingly, adults were rarely mentioned except when in the role of a predatory older male.

Some of the stories describe the decision of not drinking alcohol as a part of being responsible. The stories capture the quagmire the young people feel they are stuck in as alcohol starts entering their friendship groups’ activities at an age, they consider too young to drink alcohol:

She didnt want to explain why she chose not to drink (…). Before she had considered those who drank alcohol as troubled teenagers but now, she was watching her best friends getting drunk. (M)

A number of the stories made a contrast between being responsible and irresponsible. Some of the stories featured young people deciding they could have fun without drinking and that it was preferable because they would know what they were doing: “They had a good time despite (not drinking) (…) and didn't need alcohol to have fun, they were more aware of what they were doing and thinking (F)”. This is in line with recent studies that show how young people are actively questioning whether alcohol is integral to having fun (Törrönen et al., 2019). The characters described as responsible were also portrayed as strong because they did not crave the appreciation of their peer groups. There is this implication that they are more mature: “… they thought that by drinking they would get this appreciation and courage (F)”.

In the extract below, the story characters were described as making informed decisions about whether to drink or not. The stories also recognised how consumption of alcohol needed to be learned so that appropriate boundaries could be set. Inexperienced (younger) drinkers were at a disadvantage as they had not learned to drink “responsibly”. In the extract below, the author of the story set out who was a responsible character in the story and what that entailed. The story also outlined how one of the characters was learning how to handle her drinking so that she would not overdo it:

Selma is the responsible one and doesn’t really think it’s necessary to drink, María is not irresponsible, but maybe she doesn’t know how to drink yet, Anna thinks it’s fun to drink but doesn’t always and is often the group’s party mom (she cares about them, but mostly María as she is usually drunk)… [she] likes to drink, and may drink too much, but has started to learn the limits and feel it is not necessary to drink too much.

In the extract above, one of the characters takes care of the others. A common strategy described was having one sober friend around who was the designated driver. These strategies demanded work in the form of vigilance especially around characters who were young women. The stories described how the female characters could not fully enjoy themselves unless they had their network of predominantly female friends set up. This tied in with the fear of sexual assault from strangers. These ideas about strategies to reduce harm resonated with findings from Conroy & MacLean (2019) study. Friends, especially in stories about young women, were committed to looking out for one another: “We still looked out for one another, and all went together home in a taxi”. (F)

Some of the stories described how the story characters who did not consume alcohol felt burdened with the task of always having to look out for friends who drank irresponsibly and had to be taken home. The stories described annoyance of being taken for granted and always being the one having to take friends home safely:

At last, I found you hanging out with your other friends who by the way DRINK and you didn’t even look at me. No, no I don’t drink and therefore I am too lame and no fun having that sort of person around. I waited an hour for you until I gave up and went up. I still made sure you all had money for taxi because that is what sober friends obviously do. (F)

Part of acting responsibly involved being able to foresee possible dangers. Some stories described how story characters entered a scene where they were too late or were not sure they were reading the situation correctly. These stories described distress and not being sure how to react to a stressful situation and involved characters of all genders:

“We can’t pretend that nothing happened” (…) The memory was burned into their brain even though they (the boys) had all been drinking. They saw the young girl in the white skirt every time they blinked (…) They had fun until they went down to the basement and saw the girl on the mattress and the boy who stood by her. They were shaken up, and the fun was over. (F)

What this extract draws out is how quickly things can go wrong. A potentially fun evening turns into a nightmare. It was not only the young woman character who was victimised in this story, but also the young men who were consumed with guilt and regret. One possible explanation is that they felt unable to step into the masculine role of the hero who protects and saves women (Mann, 2012). Here, knowledge and affect were interlaced in ideas about gendered harm and gallantry.

## Discussion

The value of the young people’s stories in this study lies in how they used their knowledge and sociocultural experiences to create scenarios for their story characters who were at the same age as themselves. We assume that the stories reflect the young people’s knowledge and lived experiences of how gender features in their stories about alcohol consumption, risks in their social lives, and perceptions of shame and responsibility. The social role assigned to alcohol is present in many of the stories with emphasis on excitement and risks that were especially focused on gendered bodies and relations between young men and women. The story characters were still finding their feet as drinkers or figuring out whether drinking fits at all in their lifestyles. The different ways in how especially young women's drinking was represented depending on the level of intoxication is also of particular significance for the young people who want to drink but have not learned how to calibrate their drinking to maintain desirable level of intoxication.

Alcohol is sometimes presented as an enabler that can open vistas of experience, such as in relation to sexuality, but can also deter agency. There was a clear heteronormative and gendered undertone in the stories. We were however surprised that only one story depicted gender and sexualities outside the usual binaries. Fun experiences included both young men and women, but stories about serious experiences tended to position young women as victims and were tinged with fear. Our results are in line with research findings (Bailey et al., 2015; Griffin et al., 2013) that point out paradoxes in representations of young drunk femininity. On the one hand, young women were empowered and pleasure seeking; on the other hand, their bodies and sexualities were constituted in terms of vulnerability. This made femininity a particularly anxious space. In contrast, descriptions of heavily intoxicated young men were grotesque but deemed as either foolish because they were potentially a danger to themselves, or presented in humorous way. Different emotions were attached to different bodies, and the moral of the stories drew on ideas about respectability and regrets.

There were obviously conflicting discourses in the stories, especially about the value of drinking, with signs that young people questioned whether alcohol would always create the optimal affective mood for fun activities. Nonetheless, alcohol consumption seemed to be the norm, despite the legal age for drinking being 20 years old in Iceland. We reflected on the stories indicating the difficulty of being an active non-drinking participant of night-time recreational activities. The role of always being the friend who looked out for and drove home drunk friends was also not particularly appealing.

We want to highlight several important aspects that attracted attention in the findings. First, the stories showed how the possibility of sexual violence preyed on young people's minds. As revealed by other studies (Jensen & Hunt, 2020), it seemed to be the young women's responsibility to organise exit strategies, such as by always taking along one sober friend.

Second, the stories hardly featured any adults. Parents simply did not figure as part of the scenario in the stories, which is remarkable considering that expert advice on how to curb underage alcohol consumption in Iceland highly focuses on parental engagement (Arnarsson et al., 2018). When mentioned at all, it was related to the urgency of parents not knowing what had happened. The featured adults in the stories were represented as dangerous strangers creeping around the young women. Rolando et al. (2014) also found that young people in Finland perceive distinct boundaries between youth and adult drinking habits, leading them to develop their own youth drinking cultures characterised by reduced adult regulation and an orientation toward intoxication.

Third, there were many references to how young people needed to learn to drink alcohol responsibly (i.e., in moderation) and what great harm it could cause, especially for young women, to drink themselves senseless. The young women characters are not slut-shamed and made responsible for the violence but clearly considered more at risk and more vulnerable than young men. The participants emphasised how their story characters learned to use alcohol from their peers, especially older students, but judging by the stories, it was a tricky learning process. We wonder how knowledgeable the peer groups are in providing guidance about alcohol consumption.

Lastly, we question whetherlearning related to alcohol and drug prevention, as well as equality, is actively and systematically incorporated in primary and secondary school education. The National Curriculum of Iceland (Ministry of Education, Science and Culture, 2011) stipulates that health and well-being, along with equality, should be central components in students’ education. The Directorate of Health in Iceland (n.d.a, n.a.b) leads a project named Health Promoting Schools in Iceland and has published criteria on what works in tobacco, alcohol and drug prevention in schools. However, the schools have a choice of participating and there is no follow-up on whether the education takes place or in what form. Study findings have moreover indicated that teaching in subjects such as gender equality (Guðbjörnsdóttir & Lárusdóttir, 2017) as well as sexuality education (Sigfusdottir et al., 2020) is insufficient and unsystematic in Iceland and more diversity is needed in both topics and approaches. Also, based on our analysis, we wonder whether the existing alcohol prevention strategies in Iceland employ too much of a top-down approach, not providing space for young people’s opinions, experiences and anxieties. For example, we note how, in their stories, young people identified different risks from those outlined in policy documents, for which the main focus is on health risks. We argue that the schools, which provide an important venue for friendship groups as well as parents, should play a greater role in discussing alcohol use.

There are numerous ways to transmit alcohol and drug prevention education, but studies have shown that best practices combine transmission of knowledge and working with other competencies such as social, emotional, behavioural and moral skills (Sánchez-Puertas et al., 2022). Engaging the young people in class discussions about these issues is therefore important. Tutenges and Sandberg (2013) have pointed out how drinking stories can be used to discuss alcohol consumption in a critical way with young people. The stories collected in this research would serve as a good starting point and a way to actively engage young people in this discussion.

The freedom granted to participants by the story completion method in terms of what kinds of stories they can write has many benefits. They offered insights into the anxieties and pleasures that the participants associated with alcohol consumption. The stories seemed to act almost as outlets for many of the feelings, both positive and negative, attached to alcohol consumption. However, we also acknowledge the limitations of our data. It was difficult to convince young males to participate in the study, and their stories tended to be shorter. It is also important to analyse how young people are positioned in terms of social class, race and sexuality when it comes to the representation of alcohol consumption. We would like to explore this further in future research.
